# On Surgical Operations Performed in Health

**Published:** 1830-10-01

**Authors:** 


### XXXII.

### Surgical Operations performed at the Desire of Patients.

We find in the *Revue Médicale*, for March, 1829, the following interesting observations on this subject, by M. Paillard; they may be considered as expressive of the sentiments of M. Dupuytren. It is generally observed that severe operations, performed against the opinion of the surgeon, merely to comply with the desires of the patient, are rarely successful. Whatever precautions are taken to insure success, death often supervenes. Although the surgeon explains to the patient all the hazards of the operation, and consequently has nothing to reproach himself with, still the idea of having been the cause of the death of an unfortunate person, must painfully afflict him. The case we are about to relate, is calculated to render surgeons exceedingly circumspect, and induce them to refuse with firmness to perform operations merely to satisfy the patient. M. Dupuytren has seen the most violent symptoms supervene in consequence of the amputation of a deformed great toe. In another instance, death followed the extirpation of a supernumerary finger in an adult. A case is related of amputation of a badly-formed leg, by M. Dupuytren, which terminated fatally, and the same result took place in a case of a similar kind, operated upon by Sabatier.

The following case was communicated to Dr. Paillard by Dr. Sterlin. An old servant had been affected for some time with an ulcer of the leg which would not cicatrize permanently. Tired with being constantly obliged to attend to a disease that returned continually, he entered the Hôtel Dieu, of which Pelletan was then first surgeon, and earnestly solicited him to perform amputation. M. Pelletan at first refused, but finally yielded to the solicitations of the patient, and consented to operate, not however without previously explaining to him all the hazards he encountered; but the patient was inflexible. The first few days every thing seemed to promise a favourable termination, but quickly violent symptoms supervened, some important viscera became violently inflamed, and the patient was soon in the utmost danger. Just before his death he collected his strength, and in an energetic manner, and with an eloquence that would not be suspected in an uneducated man, he reproached M. Pelletan for his weakness in yielding to his solicitation. He died some moments after having thus given vent to his anger. M. Pelletan was of course very much affected by this painful scene, and long preserved the remembrance of it. *Amer. Journ. of Medical Sciences.*

We happen to have seen three instances of a similar kind, one of which we shall here state. During the late sanguinary and protracted warfare between France and England, it is well known that the villainous policy of Napoleon caused the prisoners of both nations to be immured for years in a wretched and mournful captivity, without hope of liberation but by death! It happened that the Editor of this Journal had charge, for a short period, of the hospital of the great depôt at Forton, near Gosport, after the death of the late Dr. O'Beirne, and the following case occurred, shortly before he took charge of the hospital, and under his own immediate observation.

One of the finest and most active of the French prisoners, a young man, who spoke English fluently, and was much employed by the English officers of the establishment, had the ring finger of one hand contracted at right angles, the result of an accident which occurred before his captivity. This crooked finger was to him a great source of annoyance, and he repeatedly solicited Dr. O'Beirne to amputate it. The Doctor, who was a very sensible and shrewd surgeon, declined the operation for a long time; but, at length, the importunities of the Frenchman prevailed, and the finger was adroitly amputated in the presence of the Editor of this Journal. Every proper precaution was taken, in respect to diet and medicine; but, in a few days after the operation, irritative fever came on, and this fine young man died!

Two other instances have occurred, of nearly a similar kind, within the observation of the writer, and the whole have impressed him with a strong aversion to operations performed in health for the removal of deformities. A young lady, of distinguished beauty and accomplishments, had, within these few years, one of her toes amputated by Sir A. Cooper, in the presence of the writer. She narrowly escaped tetanus. This was for a deformity, which prevented dancing!

---

XXXIII.

